# Highlighting some of the challenges COVID-19 has posed to the European Convention on Human Rights

**DOI:** 10.1192/bjb.2020.64

**Published:** 2020-08

**Authors:** Oluwatoyin A. Sorinmade

**Affiliations:** Consultant Older Adults Psychiatrist, Interpersonal Therapist, Course Director s12/AC/DoLS Refresher Courses, Oxleas NHS Foundation Trust, UK and Mediator, The Civil Mediation Council, UK; email: oluwatoyin.sorinmade@nhs.net

The European Convention on Human Rights (ECHR) came into force in 1953 to guarantee specific rights and freedoms for people in countries that belong to the Council of Europe and to protect their human rights and prohibit unfair and harmful practices.^1^

COVID-19 took the world by storm and invaded all aspects of humanity. The ‘normal approach’ we had to life and issues relating to everyday living changed. New norms/ways of thinking as well as laws^2^ emerged to tackle the sweeping public health crisis with consequent implications for accepted rights and freedoms under the ECHR.

Governments’ world over (including Europe) variously imposed lockdown measures to limit the spread of the disease and subsequently introduced schemes to alleviate the financial difficulties imposed on their populace and pioneered schemes such as volunteer services to help the less able with shopping, collecting medication from pharmacies etc, to lessen the hardship(s) imposed by lockdown measures.

The powers available to public authorities under Article 5(1)(e) of the ECHR to lawfully detain people for the prevention of the spreading of infectious diseases were imposed by governments across Europe with the consequence that health and law enforcement agencies acquired powers to confine otherwise healthy individuals to their homes and to isolate and screen individuals suspected to have contracted COVID-19 using powers under Article 5(1)(b) of the ECHR.^3^

The promotion of powers under Article 5(1)(e) of the ECHR did not seem to have been extended to the promotion of provisions of Article 5(4) of the same convention, which allows for everyone deprived of their liberty by arrest or detention to be entitled to take proceedings by which the lawfulness of their detention shall be decided speedily by a court and their release ordered if the detention is not lawful.

The exigencies of COVID-19 especially in the early stages meant that health resources were diverted to combat/contain the pandemic with the consequent effect that the care of some individuals were delayed and, in some instances, cancelled, for example clinics and operations, possibly leading to death as an indirect consequence of COVID-19 and interference with Article 2 rights of these individuals.

There were concerns about issues relating to the blanket approach taken to writing ‘DNAR’ (do not resuscitate) on the notes of older adults and people with intellectual disabilities without proper consultation^4^ in disregard of their Article 8 rights.^5^

There were also problems with the supply and availability of personal protective equipment^6^ to health and care professionals, with consequence on the human rights of workers^7^ to be protected from toxic exposures at work.^8^

There were concerns that the exigencies of the pandemic had the potential to cause doctors to consider factors such as the availability and capacity of current resources^9^ when making decisions about whether to continue life-saving treatment on an individual with a potential scenario where an individual could be deprived of continued treatment, consequently interfering with their Article 2 rights.^10^

A Court of Protection judgment^11^ considered the interface between the right to private and family life (Article 8), right to liberty and security (Article 5) and right to life (Article 2) among others, and drew out the primacy of absolute rights under ECHR such as Article 2 rights over qualified and limited rights (Article 8 and Article 5 rights, respectively).

Patient consultation via telephone and video calls has grown in popularity and acceptance^12^ with possible implications for Article 8 rights of patients. The effect of the lockdown on the physical and mental health of the populace as well as mortality rates, including suicides will become apparent as time goes on.

Time will tell with regard to whether the exigencies of COVID-19 had an impact on the speed of hospital discharge as well as the care of people in care homes and the care and survival of the less able and whether this interfered with their Article 2, Article 3 and Article 8 rights.^13^

Although there is the possibility under Article 15 of the ECHR for governments, in time of war or other public emergency threatening the life of the nation, to temporarily derogate from their obligation to secure certain rights and freedoms under the Convention,^14^ some of the measures put in place to tackle the exigencies of COVID-19 will endure long after the pandemic has abated in the UK – otherwise we would have learnt nothing from the pandemic and forgotten nothing. There is therefore the need, going forward, to carefully calibrate the fine balance between public health needs and safety with human rights.

The understanding is that the European Charter of Fundamental Rights that brings together the fundamental rights of everyone living in the European Union, including the rights protected by the ECHR (as brought into UK law by the Human Rights Act) will stop having effect in the UK after the UK leaves the European Union. The government has however guaranteed UK's continued commitment to ‘respect the framework’ of the ECHR.^15^

## References

[1] Equality and Human Rights Commission. What is the European Convention on Human Rights? Equality and Human Rights Commission, 2017. Available from: https://www.equalityhumanrights.com/en/what-european-convention-human-rights.

[2] Coronavirus Act 2020.

[3] *McVeigh and Others v. United Kingdom (Rep.)* [1981] no. 8022/77.

[4] Ravel P. Whose life to save? Investigating the ‘do not resuscitate’ form coronavirus controversy. Bristol Cable 2020; 10 April. Available from: https://thebristolcable.org/2020/04/bristol-coronavirus-dnr-whos-life-to-save-do-not-resuscitate-forms/#article-top.

[5] *R (Tracey) v Cambridge University Hospitals NHS Foundation Trust & Anor* [2014] EWCA Civ 33.

[6] Connolly K. German doctors pose naked in protest at PPE shortages. Guardian 2020; 26 April. Available from: https://www.theguardian.com/world/2020/apr/27/german-doctors-pose-naked-in-protest-at-ppe-shortages?CMP=Share_iOSApp_Other.

[7] Burden E. Inside Britain's PPE shortages: was the UK really so ill-equipped? Telegraph 2020; 19 April. Available from: https://www.telegraph.co.uk/business/2020/04/19/inside-britains-ppe-shortages-uk-really-ill-equipped/.

[8] *Brincat and Others v Malta* [2014] application nos. 60908/11, 62110/11, 62129/11,62312/11 and 62338/11.

[9] United Nations. Principles on Human Rights and the Protection of Workers’ Rights from Exposure to Toxic Substances, United Nations Human Rights Council, Forty second session, A/HRC/42/4. United Nations, 2019.

[10] Medical Protection Society. Emergency Law Needed to Protect Doctors at Risk of Legal Challenge when Treating COVID-19 Patients. Medical Protection Society, 2020. Available from: https://www.medicalprotection.org/uk/articles/emergency-law-needed-to-protect-doctors-at-risk-of-legal-challenge-when-treating-covid-19-patients.

[11] *Oyal vs Turkey* [2010] application no.4864/05.

[12] *BP v Surrey County Council & Anor* [2020] EWCOP 17.

[13] Attend Anywhere. *Make Travel Optional.* Attend Anywhere, no date. Available from: https://www.attendanywhere.com/.

[14] *Volintiru vs Italy* [2013] application no. 8530/08, see also [2011] UKSC 33.

[15] European Court of Human Rights. *Factsheet – Derogation in Time of Emergency*. European Court of Human Rights, 2020. Available from: https://www.echr.coe.int/Documents/FS_Derogation_ENG.pdf.

[16] Dawson J. How might Brexit affect human rights in the UK? Insights from the New Parliament 2019; 17 Dec. Available from: https://commonslibrary.parliament.uk/brexit/how-might-brexit-affect-human-rights-in-the-uk/.

